# Population structure and diversity of an invasive pine needle pathogen reflects anthropogenic activity

**DOI:** 10.1002/ece3.1200

**Published:** 2014-09-04

**Authors:** Irene Barnes, Michael J Wingfield, Ignazio Carbone, Thomas Kirisits, Brenda D Wingfield

**Affiliations:** 1 Department of Genetics, Forestry and Agricultural Biotechnology Institute (FABI), University of PretoriaPretoria, 0002, South Africa; 2 Department of Plant Pathology, Center for Integrated Fungal Research, North Carolina State UniversityRaleigh, North Carolina, 27695; 3 Department of Forest and Soil Sciences (DFS), Institute of Forest Entomology, Forest Pathology and Forest Protection (IFFF), University of Natural Resources and Life Sciences, Vienna (BOKU)Vienna, Austria

**Keywords:** Climate change, fungi, gene flow, mating type genes, *Mycosphaerella pini*, population genetics

## Abstract

*Dothistroma septosporum* is a haploid fungal pathogen that causes a serious needle blight disease of pines, particularly as an invasive alien species on *Pinus radiata* in the Southern Hemisphere. During the course of the last two decades, the pathogen has also incited unexpected epidemics on native and non-native pine hosts in the Northern Hemisphere. Although the biology and ecology of the pathogen has been well documented, there is a distinct lack of knowledge regarding its movement or genetic diversity in many of the countries where it is found. In this study we determined the global population diversity and structure of 458 isolates of *D. septosporum* from 14 countries on six continents using microsatellite markers. Populations of the pathogen in the Northern Hemisphere, where pines are native, displayed high genetic diversities and included both mating types. Most of the populations from Europe showed evidence for random mating, little population differentiation and gene flow between countries. Populations in North America (USA) and Asia (Bhutan) were genetically distinct but migration between these continents and Europe was evident. In the Southern Hemisphere, the population structure and diversity of *D. septosporum* reflected the anthropogenic history of the introduction and establishment of plantation forestry, particularly with *Pinus radiata*. Three introductory lineages in the Southern Hemisphere were observed. Countries in Africa, that have had the longest history of pine introductions, displayed the greatest diversity in the pathogen population, indicating multiple introductions. More recent introductions have occurred separately in South America and Australasia where the pathogen population is currently reproducing clonally due to the presence of only one mating type.

## Introduction

Biological invaders are plants, animals, invertebrates, or microorganisms that have become established in a new area and that threaten, or have a detrimental effect on the biodiversity and ecology of the new environment (Pimentel et al. 2000; Sakai et al. 2001; Allendorf and Lundquist 2003; Anderson et al. 2004). It is well recognized that biological invasions by fungal plant pathogens have had huge impacts on natural and managed forest ecosystems as well as plantation forests (Elton 1958; Desprez-Loustau et al. 2007). Two well-known examples of devastation caused by introduced pathogens on native trees in natural forests include Chestnut blight caused by *Cryphonectria parasitica* in North America and Europe (Anagnostakis 1987; Heininger and Rigling 1994) and Dutch elm disease caused by *Ophiostoma ulmi* and *O. novo*-*ulmi* in Europe and North America (Brasier 1991). Human-created environments such as plantation forests, consisting of non-native trees, have also been severely damaged by diseases, particularly in the tropics and Southern Hemisphere (Wingfield et al. 2001; Wingfield 2003).

The ascomycete fungus, *Dothistroma septosporum* (teleomorph: *Mycosphaerella pini*), that causes Dothistroma needle blight (DNB), is by far the most important invasive pathogen of non-native pine species (Gibson 1972; Ivory 1987; Bradshaw 2004; Barnes et al. 2008a). This disease results in successive needle defoliation, a reduction in stem diameter and height growth and, in severe cases, tree death (Gibson et al. 1964). DNB has been reported from more than 63 countries worldwide, infecting over 82 different species of pine and occasionally other conifer species in their native and non-native ranges (Bednářová et al. 2006; Watt et al. 2009).

One of the main factors contributing to the increase in biological invasions by fungal plant pathogens is the expansion of global travel and trade. This has promoted the anthropogenic introduction and spread of these organisms within and between countries, mainly via infected plant material (Richard and Lonsdale 2001; Rossman 2001; Wingfield et al. 2001). In the Northern Hemisphere, for example, planting stock of *Pinus nigra* and *Pinus mugo* infected with *D. septosporum* was intercepted when transported into the Czech Republic via Hungary (Jankovský et al. 2004). The intercontinental spread of *D. septosporum* was most likely due to the increase in air traffic and the establishment of non-native commercial pine plantations in the Southern Hemisphere, especially after World War II (Gibson 1972). In New Zealand, the pathogen is speculated to have been introduced by forestry officials who travelled to East Africa in 1957 to observe DNB (Hirst et al. 1999). The pathogen was discovered 5 years later causing disease in the central North Island forests of New Zealand (Gilmour 1967).

Introductions into new environments can also be due to natural events such as long distance dispersal via wind-blown spores (Brown and Hovmøller 2002; Stukenbrock et al. 2006). Natural long distance dispersal of *D. septosporum* into Australia from New Zealand via spores in mist clouds blown over the Tasman Sea, has been suggested by Edwards and Walker (1978). This view is supported by the fact that strict quarantine regulations are implemented in Australia, which would make it unlikely that an introduction via plant material had occurred (Edwards and Walker 1978; Bradshaw 2004).

A characteristic of a successful invasive species, after introduction, lies in its ability to become established in a new environment and then to spread to new areas (Sakai et al. 2001). Short distance spread of the spores of *D. septosporum*, either as asexual conidia or sexual ascospores is very effective (Gibson 1972; Bradshaw 2004). The ability of the pathogen to expand its range can thus be observed from the chronology of the first records from countries both in the Northern and the Southern Hemisphere.

In the Southern Hemisphere, the first report of *D. septosporum* was from Zimbabwe in 1940, where young *Pinus radiata* trees were severely damaged (Table S1, Barnes 1970; Gibson 1972). In 1957, the pathogen was found in Tanzania and within 7 years, the associated disease had spread to all major *P. radiata* plantations in Kenya, Malawi, and Uganda (Gibson 1972). Similarly, in Chile and New Zealand, where 92% of the world’s *P. radiata* is grown (Toro and Gessel 1999; Rogers 2002), *D. septosporum* has caused disease epidemics since 1957 (Gibson 1972) and 1963 (Gilmour 1967), respectively. In Australia, the pathogen was found much later, in 1975 (Edwards and Walker 1978).

During the past 50 years, *D. septosporum* has successfully invaded and become established in many European countries. During this time, the geographic range of the pathogen has expanded and serious disease epidemics have emerged. In Serbia, for example, the pathogen has been known since 1955 (Krstić 1958) and by 1988, severe epidemics on both native and exotic hosts had occurred (Karadzić 1988). From there, the pathogen spread northwards, and by 1969 it had entered the southern part of Hungary (Karadzić 1989). By 1995, the DNB fungus had spread to virtually all *P. nigra* monocultures in Hungary (Szabó 1997), most of which had been established during a pine afforestation program in the 1960s (ÁESZ 2002). Approximately 2 years later, the disease was recorded in the Southern tip of Slovakia, close to the border of Hungary (Barnes et al. 2011). Today, it is found throughout Slovakia on both native and non-native pine species (Zúbrik et al. 2006).

Given its importance, it is surprising how little is known regarding the origin of *D. septosporum*. In this regard, there are two hypotheses (Evans 1984; Ivory 1994). Based on its presence on native pines in the high elevation, minimally disturbed cloud forests of Central America, and in the absence of conspicuous epidemics, one view is that *D. septosporum* is native to that region (Evans 1984). The fact that the pathogen has also been found on indigenous pine trees in remote forests in the Himalayas prompted Ivory (1994) to suggest that it might also be native to these areas. Given the fact that two fungal species, *D. pini* and *D. septosporum* cause DNB (Barnes et al. 2004) and that these species are almost impossible to discriminate from each other without DNA-based identification techniques (Ioos et al. 2010; Barnes et al. 2011), reports such as those listed above, and hypotheses regarding the origin of these pathogens, are speculative at best. Recently, the presence of *D. septosporum* on native blue pine (*P. wallichiana*) trees in the Himalayas has, however, been confirmed (Barnes et al. 2008a) and this area of origin seems probable.

Although the biology and ecology of *D. septosporum* has been well documented, little is known regarding the global structure and population genetics of the pathogen. The aim of this study was to determine the diversity and genetic structure of *D. septosporum* populations from a broad collection of isolates from 14 countries across six continents using 12 polymorphic microsatellite markers. More specifically, the aim was to determine whether the patterns of genetic diversity and structure of *D. septosporum* populations reflect the movement of its hosts from the Northern Hemisphere to the Southern Hemisphere or whether epidemic populations on non-native pines in both the Northern and Southern Hemisphere reflect recent introduction events. In addition, the degree of genetic differentiation and variation that exists between populations and groups of populations was determined and gene flow between geographic locations was estimated. For the possibility of sexual recombination to occur, both mating types need to be present in a population. Thus, the frequency and distribution of mating types in the *D. septosporum* populations was also determined.

## Materials and Methods

### Sampling, fungal isolations, and DNA extraction

Isolates of *D. septosporum* were obtained from a variety of *Pinus* spp. representing 14 different countries (referred to as populations) from six regions (=continents). These included Africa (South Africa and Kenya), Europe (Austria, Czech Republic, Hungary, Poland, Romania, Slovakia), Asia (Bhutan), North America (U.S.A.), South America (Chile and Ecuador), and Australasia (Australia and New Zealand) (Table 1). The main pine species from which collections were made included *P. nigra* in the Northern Hemisphere and *P. radiata* in the Southern Hemisphere. The sampling strategy from plantation forests included collecting a handful of diseased needles from every second tree along two or more transects. Samples from native pine species, or from hosts not growing in plantations were opportunistically collected in areas where infected trees could be located. Samples were collected at one or several locations in a country (Table 1).

**Table 1:** Isolates of *Dothistroma septosporum* from different countries and hosts used in this study

Country	Locality	*Pinus* host	Native or introduced host in country	No. of isolates	Date collected	Collectors
Austria	Hollenstein/Ybbs, province Lower Austria	Planted *P. sylvestris*	Native	5	July 2004	T. Kirisits, T. L. Cech, M. Brandstetter
		Planted *P. mugo*	Native	5	July 2004	T. Kirisits, T. L. Cech, M. Brandstetter
		Planted *P. nigra*	Native	2	July 2004	T. Kirisits, T. L. Cech, M. Brandstetter
		Planted *P. cembra*	Native	1	July 2004	T. Kirisits, T. L. Cech, M. Brandstetter
	Vienna, forest experimental garden, “Knödelhütte”, BOKU	Planted *P. flexilis*	Introduced	4	April 2004	T. Kirisits
	Thenneberg, province Lower Austria	Native *P. nigra*	Native	27	June 2004	T. Kirisits, H. Konrad
		Native *P. nigra*	Native	4	June 2006	T. Kirisits
	Near Wr. Neustadt, province Lower Austria	Native *P. sylvestris*	Native	1	June 2006	T. Kirisits
	Raumberg, province Styria	Planted *P. mugo*	Native	2	June 2006	T. Kirisits
	Gstatterboden, national park “Gesäuse”, province Styria	Native *P. sylvestris*	Native	8	June 2006	T. Kirisits
		Native *P. mugo*	Native	2	June 2006	T. Kirisits
Czech Republic	Tisnov, Riegrova road	Planted *P. nigra*	Native	1	July 2006	L. Jankovsky
	South Moravia, Lanzhot, Forest district Lanzhot	Native *P. nigra*	Native	12	July 2006	L. Jankovsky
Hungary	Sopron, botanical garden of the University of West Hungary	Planted *P. mugo*	Native	5	August 2005	T. Kirisits
	Near Diszel	Planted *P. nigra*	Native	45	May 2007	I. Barnes
Poland	Miechów Forest District, Goszcza Forest Unit, near Cracow	Planted *P. nigra*	Native	34	June 2003	T. Kowalski
Romania	Valea Putnei (near fishing pond), Suceava	Native *P. nigra*	Native	4	July 2007	G. Hoch
Slovakia	Strazovske vrchy, valley between Zliechov and Kosecke Podhradie	Native *P. nigra*	Native	24	July 2006	L. Jankovský
Bhutan	Yusipang, Thimphu dzongkhag	Planted *P. radiata*	Introduced	3	July 2005	T. Kirisits, M. J. Wingfield, D. B. Chhetri
	Ura, Bumthang dzongkhag	Native *P. wallichiana*	Native	1	May 2005	H. Konrad, D. B. Chhetri
	Tangsibi, Bumthang dzongkhag	Native *P. wallichiana*	Native	6	July 2005	T. Kirisits, M. J. Wingfield, D. B. Chhetri
	Lamey Goemba, Bumthang dzongkhag	Native *P. wallichiana*	Native	2	July 2005	T. Kirisits, N. Gyeltshen
USA	Lochsa Historical Ranger Station, Idaho	Native *P. ponderosa*	Native	1	2004	L. M. Carris
	Missoula Lola National Forest, Montana	Native *P. contorta v. latifolia*	Native	9	May 2006	D. Six
Kenya	Napkoi	Planted *P. radiata*	Introduced	11	Jan 2001	J. Roux
South Africa	Hogsback, Eastern Cape	Planted *P. radiata*	Introduced	73	Aug 2001	J. Roux
(RSA)	Haenertsburg area, Tzaneen, Limpopo	Planted *P. radiata*	Introduced	27	Sep 2002	I. Barnes
Chile	Canteras plantation, Bio Bio, VIII Region	Planted *P. radiata*	Introduced	27	2001	M. J. Wingfield
	Canteras plantation, Bio Bio, VIII Region	Planted *P. radiata*	Introduced	1	2007	R. Ahumada
	Dollinco, Valdivia, X Region	Planted *P. radiata*	Introduced	23	2001	M. J. Wingfield
	Naguilan, Valdivia, X Region	Planted *P. radiata*	Introduced	8	2001	M. J. Wingfield
Ecuador	Lasso Highlands, Cotopaxi	Planted *P. muricata*	Introduced	17	2001	M. J. Wingfield
Australia	Canberra (A.C.T)	Planted *P. radiata*	Introduced	6	2000	K. Old
	State Forests of New South Wales in Tumut	Planted *P. radiata*	Introduced	35	June 2003	A. J. Carnegie
New Zealand	Karioi	Planted *P. ponderosa*	Introduced	1	Aug 2001	M. Dick
	Karioi	Planted *P. contorta*	Introduced	1	Aug 2001	M. Dick
	FRI Nursery, Rotorua	Planted *P. radiata*	Introduced	3	Aug 2001	M. Dick
	Hokonui Forest	Planted *P. ponderosa*	Introduced	1	Aug 2001	M. J. Wingfield
	Lake Okareka, Rotorua	Planted *P. radiata*	Introduced	1	Aug 2001	M. J. Wingfield
	Kaingora Forest	Planted *P. radiata*	Introduced	15	Feb 2003	M. J. Wingfield

Isolations were made from a single conidiomata on a needle, per tree, as described by Barnes et al. (2004). Cultures were grown on 2% malt extract agar (MEA, Biolab, Midrand, Johannesburg) at 20°C. All cultures are maintained in the culture collection (CMW) of the Forestry and Agricultural Biotechnology Institute (FABI), University of Pretoria, South Africa. Genomic DNA was extracted from each isolate using freeze-dried, ground mycelium, from 2- to 3-month-old cultures with the aid of the DNeasy Plant Mini Kit (Qiagen, Hilden, Germany).

### Haplotype identification

The genotypes of isolates were determined at 12 microsatellite loci including Doth_E, Doth_F, Doth_G, Doth_I, Doth_J, Doth_K, Doth_L, Doth_M, Doth_O, Doth_DS1 and Doth_DS2 (Barnes et al. 2008b), and DCB2 (Ganley and Bradshaw 2001). An internal diagnostic marker, Doth_A (Forward: CGG CAT CAC TGT TCA CCA CGC, Reverse: GAA GCC GCA AGT GCC AAT GGC), was used to confirm the identity of the isolates as *D. septosporum* during allele scoring and based on the fact that *D. septosporum* is monomorphic for allele 124 (Barnes et al. 2008b).

Polymerase chain reactions (PCR) were carried out in a total volume of 12.5 *μ*l and contained 5–10 ng DNA template, 0.06 U FastStart Taq DNA Polymerase (5 U/*μ*L) (Roche Diagnostics GmnH, Mannheim, Germany), 1× PCR buffer containing 2 mmol/L MgCl_2_, 0.25 mmol/L of each dNTP, 100 nmol/L of the forward and reverse primers, one of which was fluorescently labelled and 1 mmol/L additional MgCl_2_. The PCR conditions consisted of an initial denaturation step of 10 min followed by 10 cycles of 94°C for 30 s, specified annealing temperature for 45 s (as described in Barnes et al. 2008b) and 72°C for 1 min. A further 30 cycles were carried out using the same conditions as those described above except that a 0.5 s increment was added to the elongation time. A final elongation period of 30 min at 60°C was added to avoid the +A effect (Clark 1988; Magnuson et al. 1996) during genescan analyses. PCR amplicons were separated, with a 100 bp marker, on 2% low electroendosmosis agarose gels (Roche Diagnostics) stained with ethidium bromide and visually analyzed under UV light (Vilber Lourmat, Omni-Science, Cedex, France). PCR amplicons were not purified.

To facilitate multiplexing, PCR amplicons (for each individual) were combined according to the approximate size of the amplicons and type of fluorescent label attached to the primer. Samples were subjected to electrophoresis on an ABI 3100 sequencer. Allele assignments were determined using ABI-Prism ®GeneMapper™ software version 3.0 (Applied Biosystems, Foster City, CA). Multilocus genotypes were obtained by combining the alleles present at all twelve loci for each isolate. All isolates having the same multilocus haplotype in a population were considered as clones.

### Genetic data analyses

#### Genetic diversity

Allele frequencies were estimated for each SSR locus using the program popgene version 1.32 (http://www.ualberta.ca/~fyeh) (Yeh et al. 1999). The total number of alleles, unique alleles and gene diversity (Nei 1973), were computed for each population and region across all 12 loci.

In addition to calculating gene diversity, estimates of allelic richness were computed for each population and region in the program fstat for windows, version 2.9.3.2 (http://www2.unil.ch/popgen/softwares/fstat.htm) (Goudet 2001). Unequal sample sizes were standardized, by rarefaction, to a uniform sample size of the smallest population and region (USA/North America, *N* = 10) as described by El Mousadik and Petit (1996). Romania was excluded from this analysis due to low sample size (*N* = 4). The clonal fraction was calculated for each population by dividing the number of genotypes observed in the population by the total population size and subtracting this from one.

Measures of genotypic diversity (D) were quantified in Multilocus version 1.3 (http://www.agapow.net/software/multilocus) (Agapow and Burt 2001) as (*n* / *n* − 1)(1 − Σ_*i*_ *pi*^2^) where *pi* is the frequency of the *i*th genotype and *n* is the number of individuals sampled. Here, the multilocus genotype of all possible pairs of individuals is compared and the proportion of pairs that are different is calculated. Completely clonal populations would score a value of 0 while those where all individuals have different multilocus genotypes would score 1.

The hierarchical partitioning of molecular variation within and among populations and among regions was assessed with an AMOVA -test implemented in genalex version 6.1 (Peakall and Smouse 2006) using the complete dataset. The significance was tested by 1000 permutations of the dataset. Only regions with more than two populations were included in the analyses. The null hypothesis of no genetic difference between populations was rejected at *P* < 0.05.

#### Population structure

The genetic distance between all isolates was calculated using Nei’s standard distance *D*_*A*_ (Nei et al. 1983) in Populations version 1.2.32 (http://bioinformatics.org/project/?group_id=84). The distance matrix generated was used to construct a Neighbor-joining (NJ) tree in Mega version 5 (Tamura et al. 2011).

The program structure version 2.2 (Pritchard et al. 2000; Falush et al. 2003) was used to determine the optimal number of populations (K) and to assign individuals to these distinct populations based on their genotype data without any prior indication of geographic location or collection sites. The program uses a Bayesian Monte Carlo Markov Chain (MCMC) clustering algorithm and the simulations assumed a model of mixed ancestry and correlation of allele frequencies within clusters. Individuals were assigned to clusters to minimize Hardy-Weinberg disequilibrium and linkage disequilibrium between the loci within each cluster. The MCMC scheme was run for 100,000 iterations after a burn-in period of 10,000. Twenty simulations were performed for *K* ranging from 1 to 14 to verify the convergence of the Log likelihood values for each value of *K*. Delta *K* (Δ *K*), a statistic based on the rate of change in the log probability of data with respect to the number of clusters, was used to interpret the real number of clusters (Evanno et al. 2005). After the optimal *K* was determined, a final parameter of 1 million MCMC replicates and a burn-in period of 100,000 was run for the assignment of individuals into *K* populations.

### Multidimensional scaling using principal components

Principal Component Analyses (PCA) and their applications to control for population stratification were performed using the methods described by Patterson et al. (2006). This analysis involves three steps: (1) apply PCA to microsatellite data; (2) determine the significant principal components; and (3) identify the optimal number of clusters defined as the subpopulations after correcting for population structure. Each microsatellite allele was converted to a binary allele such that for each microsatellite locus, there were as many columns generated as there were alleles at that specific locus (Patterson et al. 2006). PCA was performed on normalized data to improve resolution of underlying population structure recovered using STRUCTURE. Principal components were normalized to unit length and the number of significant axes of variation was determined using the Tracy-Widom statistic (Tracy and Widom 1994). The optimal number of distinct clusters was based on the Gap Statistic, which compares within cluster variance with that expected under a reference null distribution based on PCA (Tibshirani et al. 2001). The clustering was performed without any a priori knowledge of population units and was, therefore, an unbiased estimate of underlying population structure. The association of statistically significant clusters with geographic locality and host species was displayed graphically using scatter plots (3D) for consecutive combinations of three significant principal components, generated using the Scatterplot3D (Ligges and Mächler 2003) package in R (R-Development-Core-Team 2010). Each individual was represented by a single point in three-dimensional space and the host species, sampling locality and cluster attributes of each individual is also plotted in a different colour and symbol. All methods were implemented in a Mobyle SNAP Workbench, a web-based analysis portal deployed at North Carolina State University (Price and Carbone 2005; Aylor et al. 2006; Monacell and Carbone 2014).

### Multilocus inference of migration

An MPI version of Migrate-n Version 3.4.4 (Beerli 2006) implemented in Mobyle SNAP Workbench was used to calculate the mutation-scaled population sizes (*θ*) and the mutation-scaled immigration rates (*M*) between populations. *M* is defined as *m*/*μ*, where *m* is the proportion of migrants in the population in each generation and *μ* the mutation rate per generation per microsatellite locus. *M* therefore measures the importance of immigration versus mutation in introducing new variants into a population. The number of alleles per locus was used as a measure of mutation rate (*μ*) for the microsatellite data. Theta (*θ*), is the product of the inheritance scaler (=2 for haploids), the effective population size (*N*_*e*_), and the mutation rate (*μ*) per generation (*θ* = 2*N*_*e*_*μ*). Migrate uses a Bayesian approach based on the coalescent theory to estimate the population parameters. Microsatellite input data were converted to repeat numbers and a Brownian motion approximation to the stepwise mutation model was used. Starting values of *θ* and *M* were generated from *F*_ST_ assuming an inheritance scaler of 0.5 (for haploids). For the search strategy, the mutation rate was set to constant for all loci, the posterior distributions were generated using slice sampling and a static heating scheme with four chains of temperatures of 1,000,000, 3, 1.5, and 1 was used. Analyses were performed using one long chain, 100,000 steps recorded every 100 steps and a burn-in of 50,000. Three independent Migrate runs using different starting random seed numbers were performed on two datasets to ensure convergence of parameter estimates. The first dataset was used to compare migration between the six continents and the second between the 14 individual countries.

### Mating type distribution

The mating types *MAT1-1* and *MAT1-2* were assayed using a set of primers developed by Groenewald et al. (2007), to amplify the mating-type idiomorphs of *D. septosporum*. Degenerate primers (Dot Mat1r 5′- TTGCCTGACCGGCTGCTGGTG-3′ and Dot Mat2r 5′- CTGGTCGTGAAGTCCATCGTC-3′) and species-specific primers (*D. septo* Mat2f 5′- GTGAGTGAACGCCGCACATGG-3′ and *D. septo* Mat1f 5′- CGCAGTAAGTGATGCCCTGAC-3′) were multiplexed in a single reaction. PCR reactions were carried out in a total volume of 12.5 *μ*L and consisted of: 10–20 ng DNA, 1× PCR buffer containing 2 mmol/L MgCl_2_, 0.5 mmol/L MgCl_2_, 0.12 mmol/L of each dNTP, 200 nmol/L of each of the four primers and 0.032 U FastStart Taq DNA Polymerase (5 U/*μ*L) (Roche Diagnostics). Cycling conditions consisted of an initial denaturation of 5 min at 94°C followed by 40 cycles of 94°C for 20 s, 65°C for 20 s, 72°C for 40 s and a final elongation of 7 min at 72°C. The presence of the *MAT1-1* idiomorph was indicated by an amplicon size of 823 bp and these isolates were designated MAT 1. Presence of the *MAT1-2* idiomorph was determined by the amplification of a fragment of 480 bp and these isolates were designated MAT 2. An exact binomial test, using two-tailed *P*-values (http://udel.edu/~mcdonald/statexactbin.html) was used to determine whether populations deviated from the null hypothesis of a 1:1 ratio of mating types.

## Results

### Haplotype identification

A total of 471 isolates were successfully recovered from the infected pine needles. All isolates screened with the internal diagnostic marker, Doth_A, produced an allele size of 124 bp, consistent with *D. septosporum*. Thirteen isolates from Hungary that produced an allele size of 109 bp were identified as *Dothistroma pini* (Barnes et al. 2011) and were excluded from further analyses (Table 1). Ultimately, 458 isolates were used in the study and after clone-correction 234 haplotypes were obtained across the populations (Table 2).

**Table 2:** Summary diversity statistics of *Dothistroma septosporum* isolates within populations and regions

Continent	Country	*N*^1^	No. of haplotypes^2^	Total no. of alleles	Unique alleles	% alleles unique (from 130 total)	% loci polymorphic	*h*^3^	Allelic richness	D^4^
Northern Hemisphere										
	Austria	61	45	85	10	7.69	100	0.55	4.59	0.98
	Czech	13	13	47	3	2.31	92	0.53	3.84	1
	Hungary	50	35	61	2	1.54	100	0.52	3.89	0.98
	Poland	34	27	39	2	1.54	92	0.43	2.92	0.98
	Romania	4	3	25	2	1.54	75	0.39	N/A	0.83
	Slovakia	24	23	53	2	1.54	92	0.48	3.78	1
Europe	Total	186	146	109	47	36.15	100	0.57	4.79	1
Asia (Bhutan)	Total	12	12	36	2	1.54	67	0.33	2.97	1
North America (USA)	Total	10	7	28	8	6.15	75	0.25	2.33	0.91
Southern Hemisphere										
	Kenya	11	9	23	2	1.54	75	0.32	1.92	0.96
	RSA	100	35	30	0	0	92	0.34	2.16	0.86
Africa	Total	111	44	37	2	1.54	100	0.38	2.47	0.88
	Chile	59	11	22	0	0	42	0.22	1.59	0.83
	Ecuador	17	10	21	0	0	50	0.20	1.72	0.93
South America	Total	76	20	23	4	3.08	50	0.22	1.69	0.87
	Australia	41	4	15	1	0.77	25	0.06	1.19	0.62
	N. Zealand	22	2	13	0	0	8	0.01	1.05	0.09
Australasia	Total	63	5	16	2	1.54	33	0.05	1.20	0.53

1 *N* = Total number of isolates.

2 Equivalent to samples that have been clone-corrected.

3 *h* = Nei’s (1973) gene diversity.

4 Genotypic diversity.

### Genetic data analyses

#### Genetic diversity

From 12 microsatellite loci, a total of 130 alleles were inferred ranging from two alleles at Locus_DCB2 to 24 alleles at Locus_L. The allele frequencies at each locus were recorded for all populations in Table S2 (Supporting information). Other indices of variation are reported in Table 2 for each population (country) and region (continent).

Isolates from Austria had the highest percentage of unique alleles at 7.7% (based on a total of 130 in the entire data set) followed by the United States (6.2%). Only the Austrian and Hungarian populations showed 100% polymorphism for the 12 loci and contained the highest allelic richness (4.59 and 3.89, respectively). In the Southern Hemisphere, although two unique alleles were present in the Kenyan population, those from South Africa, Chile, Ecuador, and New Zealand contained no unique alleles (Table 2). Gene diversity per country ranged from a high value of 0.55 in Austria to a low value of 0.01 in New Zealand. Similarly, allelic richness ranged from 4.59 in Austria to 1.05 for New Zealand.

At the continental scale, the European population had the greatest number of alleles (109), percentage of unique alleles (36.2%), gene diversity (0.57) and allelic richness (4.79). The North American, Asian, and African populations contained moderate levels of diversity, with values of 6.2/0.25/2.33 and 1.5/0.33/2.97 and 1.5/0.38/2.47, for the percentage of unique alleles, gene diversity and allelic richness, respectively. The South American and Australasian populations had the lowest levels of diversity with gene diversity and allelic richness values of 0.22/1.7 and 0.05/1.2, respectively. The populations from South America, however, contained the third highest number of unique alleles at 3.1%, after Europe and North America. Overall, the clonal fraction in the Northern Hemisphere populations was low (mean 17%), compared to those in the Southern Hemisphere (mean 65%) (Table 3).

**Table 3:** Clonal fraction and mating types observed within populations of *Dothistroma septosporum* from 14 countries

			Exact binomial test			
			Observed		Expected	
Countries	Clone-corrected sample size	Clonal fraction	MAT1-1	MAT1-2	MAT1-1/2	*P*-value (two-tailed test)
Austria	45	0.26	29	31	30	0.897
Czech	13	0.00	6	7	6.50	1.000
Hungary	35	0.30	24	23	23.50	1.000
Poland	27	0.21	9	24	16.50	0.014*
Romania	3	0.25	4	0	2	0.125
Slovakia	23	0.04	11	12	11.50	1.000
Bhutan	12	0.00	5	7	6	0.774
USA	7	0.30	9	1	5	0.021*
Kenya	9	0.18	8	3	5.50	0.227
RSA	35	0.65	39	61	50	0.035*
Chile	11	0.81	0	59	29.50	0*
Ecuador	10	0.41	0	17	8.50	0*
Australia	4	0.90	0	41	20.50	0*
New Zealand	2	0.91	0	22	11	0*

* Mating type departs from the null hypothesis of a 1:1 ratio at *P* < 0.05.

The collection of isolates from the Czech Republic and Bhutan contained no duplicate haplotypes. The highest clonal fraction was observed for the New Zealand population at 91%. Genotypic diversities were higher for collections from Northern Hemisphere countries, ranging from 0.83 to 1 (Table 2). The genotypic diversity for isolates from the Southern Hemisphere ranged from 0.09 (New Zealand) to 0.96 (Kenya). All but four multilocus haplotypes were unique to countries. The exceptions included those present in isolates from both Chile and Ecuador and one that was present in both New Zealand and Australian isolates.

AMOVA analyses showed that at a global scale, with the continents representing regions, most of the variation was distributed within populations (61%), but a significant proportion of the variation (32%) was also attributed to differences among regions (Table 4). Eight percent of the variation was partitioned among populations within regions. AMOVA analyses for individual regions showed that for both the South American and Australasian collections, a high percentage of the variation was within populations (99% and 96%, respectively), with no genetic differences observed among these populations (Table 4). A large proportion of the variation for the African collections was observed between Kenya and South Africa (33%, Table 4). Within Europe, although variation among populations was low (Φ-PT = 0.088, *P* = 0.001), it was still significant for population structure.

**Table 4:** Analyses of molecular variance (AMOVA) of *Dothistroma septosporum* within populations, among populations and between regions

			% Variation							
Dataset	Number of populations	Number of regions	Among Regions	df	Among Pops	df	Within Pops	df	ØPT- statistics	*P*- value^1^
Europe	6	1	–	–	9%	5	91%	141	0.088	0.001^2^
Asia	1	–	–	–	–	–	–	–	–	–
North America	1	–	–	–	–	–	–	–	–	–
Africa	2	1	–	–	33%	1	67%	42	0.333	0.001^2^
South America	2	1	–	–	1%	1	99%	22	0.006	0.319
Australasia	2	1	–	–	4%	1	96%	4	0.040	0.537
All Regions	14	6	32%	5	8%	8	61%	226	0.395	0.001^2^
Northern vs. Southern Hemisphere	14	2	11%	1	25%	12	64%	226	0.358	0.001^2^

1 *P*-value based on 1000 randomizations.

2 The null hypothesis of no population structure is rejected at *P* < 0.01.

#### Population structure

**Figure 1:**
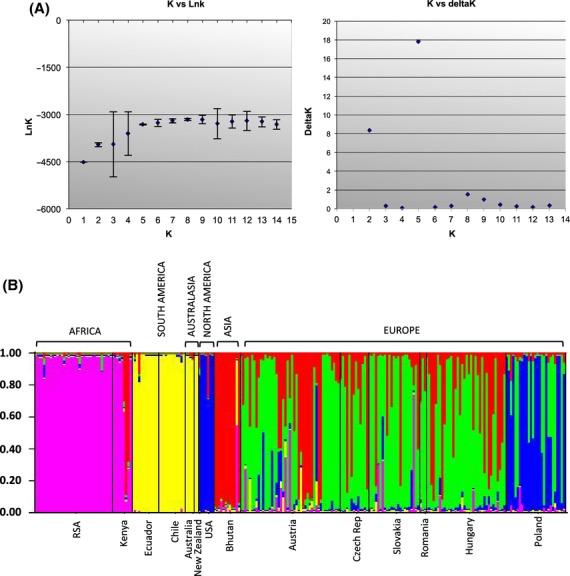
Population structure of *Dothistroma septosporum*; (A) Optimal number of populations (*K* = 5) as determined by the LnK and DeltaK values obtained from structure analyses using the admixture model with correlated allele frequencies; (B) Estimated clustering of *D. septosporum* isolates on a global scale based on 12 loci. The proportion of individuals within countries assigned to one of five clusters (*K* = 5) as identified by Structure is shown.

Structure analyses revealed the highest posterior probability for five clusters/populations (Fig. 1). Clusters consisted mainly of isolates from the same geographic areas of distribution. The Southern Hemisphere isolates were partitioned into two clusters. Cluster 1 comprised the African isolates from South Africa and Kenya while Cluster 2 comprised the rest of the Southern Hemisphere isolates from Chile, Ecuador, Australia, and New Zealand. For the Northern Hemisphere, three additional clusters were observed. The isolates from the United States mainly fell into Cluster 3 and those from Bhutan into Cluster 4. The European isolates contained mixed assignments, mainly into Cluster 5 (Romania, Hungary, Slovakia, Czech, Poland, and Austria), but with individuals from these countries also being assigned to Cluster 3 (mainly Poland) and a few into Cluster 4 (Romania, Hungary, Slovakia, Czech, and Austria).

**Figure 2:**
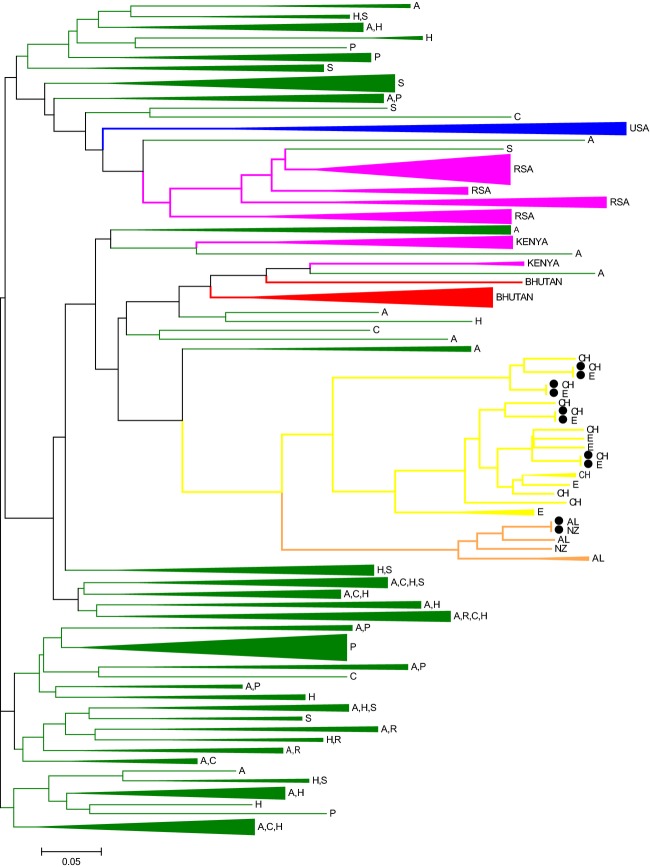
NJ tree based on Nei’s genetic distance *D*_*A*_ among isolates of *Dothistroma septosporum* on a global scale. Colours of lineages are in accordance with the populations as identified in Structure (Fig. 1 and 5). Lineages in green represent isolates from Europe (A: Austria, C: Czech, H: Hungary, P: Poland, R: Romania, S: Slovakia); blue from the United States; pink from Africa (Kenya and RSA:South Africa); red from Bhutan; yellow from South America (Ch: Chile, E: Ecuador) and those in orange represent isolates from Australasia (AL: Australia, NZ: New Zealand). Black dots indicate instances where a haplotype is shared between different countries as in the case of Chile and Ecuador and between Australia and New Zealand.

The NJ tree based on Nei’s genetic distance *D*_*A*_ showed similar clustering of isolates into distinct clades based broadly on the country and continent from where they were collected (Fig. 2). Lineages have been coloured in accordance with the geographic clusters identified in Structure (Fig. 1 and 5). Although Structure grouped the isolates from South America and Australasia into one cluster, distinct lineages can be seen here for the South American (yellow lineage) and Australasian (orange lineage) isolates (Fig. 2). In four cases, isolates from Ecuador and Chile share identical haplotypes (indicated as black dots) while isolates from Australia and New Zealand share one identical haplotype. Similarly, most of the isolates from South Africa cluster together, although genetically distinct from Kenya (both indicated in pink). The isolates from the United States form their own unique, distinct lineage (blue), as did most of the isolates from Bhutan (indicated in red). The isolates from Europe, however, showed many divergent branches and formed several subgroups (indicated in green). Isolates from Austria, Romania, Hungary, the Czech Republic, and Poland were randomly distributed throughout the tree indicating a high level of migration.

### Multidimensional scaling using principal components

**Figure 3:**
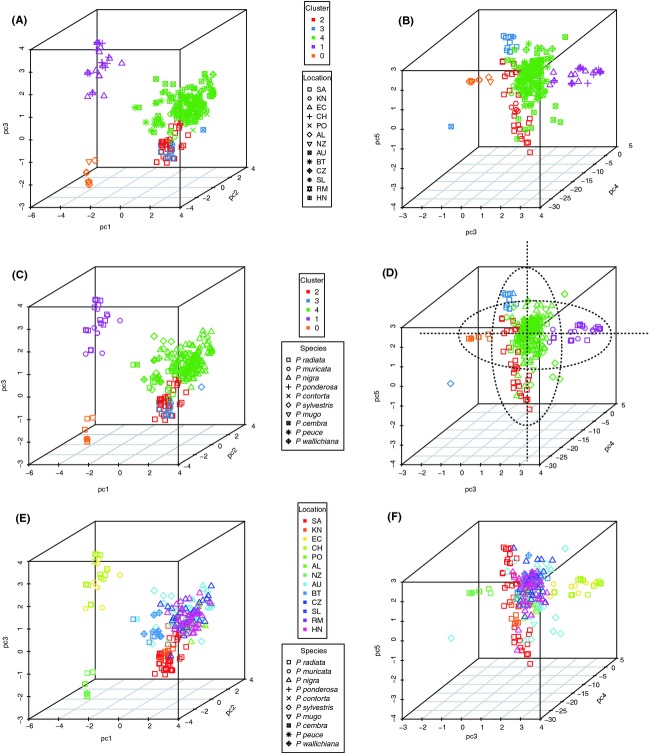
Principal component analysis of 448 individuals of *Dothistroma septosporum* from different hosts and countries. Scatter plots of principal component scores show the distribution of individuals along five significant axes of variation. Each point in the graph represents a single isolate. Plots of the first three (PC1, 2, 3) and last three (PC3, 4, 5) significant axes of variation in three-dimensional space for cluster/location (A, B), cluster/species (C, D), and location/species (E, F) are represented using different colours and symbols. Oval outlines in (D) show *P. radiata* as harboring variation at the extremes of two axes of variation, PC3 and PC5. Sampling localities are abbreviated as follow: SA, South Africa; KN, Kenya; EC, Ecuador; CH, Chile; PO, Poland; AL, Australia; NZ, New Zealand; AU, Austria; BT, Bhutan; CZ. Czech Republic, SL, Slovakia; RM, Romania, HN, Hungary.

**Figure 4:**
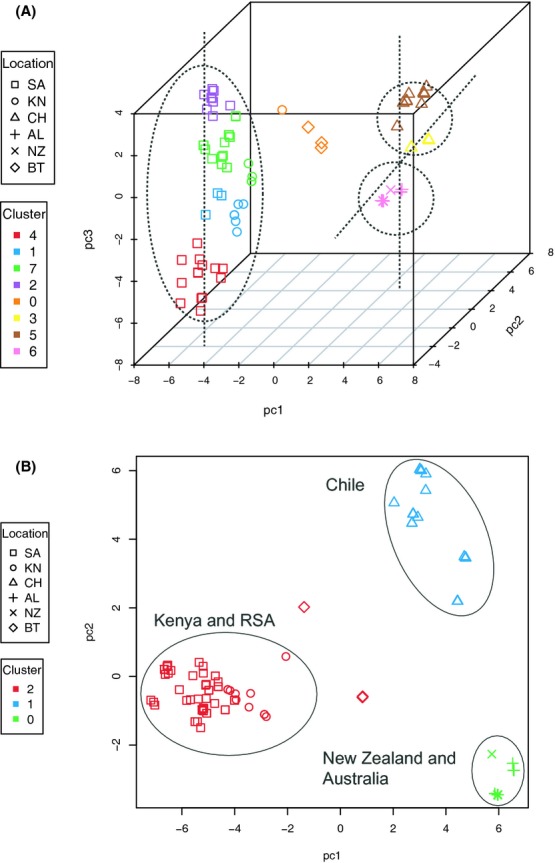
Principal component analysis for isolates of *Dothistroma septosporum* from *P. radiata*. Scatter plots show the principal component scores for three significant axes of variation in three-dimensional space (A) and the first two principal components in two-dimensional space (B) for all isolates of *D. septosporum* sampled from *P. radiata*. (A) Eight distinct clusters are inferred based on three significant axes of variation. In PC3 there are four distinct clusters from South Africa. Approximately, 22%, 12%, and 10% of the total variance is partitioned between PC1, PC2, and PC3, respectively, and separates the South African clusters from the New Zealand/Australian and Chile clusters. The latter two clusters are separated by variation in PC2 and PC3. (B) Three distinct clusters within the Southern Hemisphere are inferred if we examine only the first two principal components. Sampling localities are abbreviated as shown in Fig. 3.

Principal component and Tracy-Widom analyses revealed five significant axes of variation and five distinct clusters based on the gap statistic similar to the number of clusters inferred with structure. Plots of the first three (PC1, 2, 3) and last three (PC3, 4, 5) significant axes are presented for cluster/geographic location (Fig. 3A and B), cluster/*Pinus* species (Fig. 3C and D), and a combination of geographic location/*Pinus* species (Fig. 3E and F). Inspection of these plots revealed *P. radiata* as harboring variation at the extremes of two axes of variation, PC3 and PC5, outlined using ovals in Fig. 3D. When only the isolates from *P. radiata* were examined (Fig. 4A), a total of eight clusters based on three significant axes of variation could be distinguished. The South African isolates fell into four of these distinct clusters as distinguished by PC3 (Fig. 4A). Approximately 20% of the variation separated the South African clusters from the New Zealand/Australian and Chile clusters. The latter two clusters were further separated by variation in PC2 and PC3 (Fig. 4A). When the first two significant axes were examined alone, then three distinct clusters were observed in the Southern Hemisphere; an African cluster with South African and Kenyan isolates (cluster 2), a South American cluster with isolates from Chile (cluster 1) and an Australasian cluster with isolates from New Zealand and Australia (cluster 0) (Fig. 4B).

### Multilocus inference of migration

**Figure 5:**
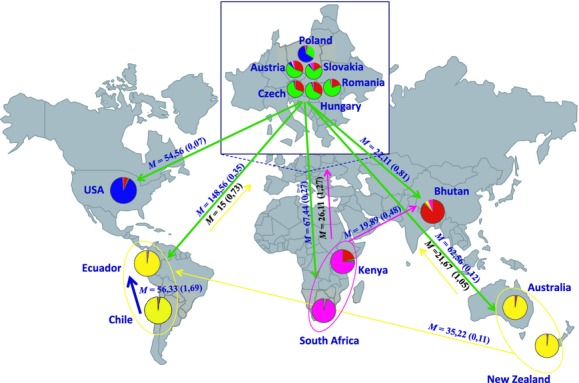
Global migration patterns and population structure of *Dothistroma septosporum*. Pie charts show the proportion of individuals in each country assigned to one of *K* = 5 groups as determined by Structure. The directional migration routes between continents, as determined by Migrate, are shown with arrows. *M* represents the mutation-scaled effective immigration rate between continents while the number of immigrants per generation (*θ*M) is indicated in parenthesis. Although Europe is the biggest donor of immigrants on a global scale, it also acts as the largest sink population. On a country level, the largest evidence of directional migration was from Chile into Ecuador (see Table 5).

A total of 36 migration parameters were tested at a continental level (6 × 6 matrix for Africa, South America, Australasia, Europe, Asia, and North America) and 196 parameters at a global scale (14 countries) in three independent runs. While there was adequate chain mixing, independent runs did not converge in estimates of the magnitude of migration among countries, or continents, for any of the runs. While absolute values did not match, in many cases similar migration directionality was observed between runs. Results of the migration parameters for the comparisons between continents for a single Migrate run are shown in Fig. 5. Migration estimates indicated that the largest donors of migrants at a global scale came from Europe and Africa. Austria and Hungary were the largest contributors for Europe and South Africa emerged as the largest donor for Africa (Fig. 5, Table 5). Europe appeared to represent the largest sink population for migrants and these were especially from the Southern Hemisphere. In the Southern Hemisphere, the most significant number of migrants was observed going from Chile into Ecuador. The sink for donors in Europe and South America was identified as Australia while New Zealand received migrants from Australia. South Africa was the largest donor of migrants to Kenya.

**Table 5:** Population estimates of theta (θ) and mutation-scaled immigration rates (*M* = m/μ) between pairs of populations of *Dothistroma septosporum* from 14 countries based on 12 microsatellite loci using MIGRATE

Source\sink^1^ populations	Theta	Austria	Czech	Hungary	Poland	Romania	Slovakia	Bhutan	USA	Kenya	RSA	Chile	Ecuador	Australia	New Zealand
Austria	0.003	–	13.00 (0–28.7)	13.00 (0–28.7)	19.67 (2–36.7)	11.67 (0–27.3)	15.00 (0–30.7)	1.00 (0–22.0)	15.67 (0–31.3)	14.33 (0–30.0)	15.00 (0–30.7)	15.00 (0–30.7)	8.33 (0–25.3)	0.33 (0–18.7)	0.33 (0–18.0)
Czech	0.096	6.33 (0–23.3)^2^	–	9.67 (0–26.0)	8.33 (0–25.3)	7.00 (0–24.0)	0.33 (0–22.0)	5.67 (0–23.3)	11.00 (0–26.7)	9.00 (0–26.0)	0.33 (0–17.3)	0.33 (0–21.3)	0.33 (0–18.0)	0.33 (0–16.7)	0.33 (0–16.0)
Hungary	0.009	12.33 (0–28.7)	11.00 (0–27.3)	–	15.67 (0–31.3)	20.33 (1.3–38.7)	12.33 (0–28.0)	3.00 (0–22.0)	16.33 (0–32.0)	0.33 (0–16.0)	3.67 (0–22.0)	11.67 (0–27.3)	5.67 (0–24.0)	9.00 (0–25.3)	2.33 (0–22.0)
Poland	0.001	7.00 (0–24.0)	9.00 (0–25.3)	0.33 (0–20.0)	–	11.00 (0–29.3)	8.33 (0–24.0)	0.33 (0–20.0)	13.00 (0–28.7)	11.67 (0–27.3)	0.33 (0–18.0)	7.00 (0–23.3)	11 (0–26.7)	0.33 (0–21.3)	5.00 (0–22.7)
Romania	0.005	0.33 (0–16.0)	0.33 (0–18.7)	0.33 (0–16.0)	0.33 (0–19.3)	–	0.33 (0–15.3)	0.33 (0–19.3)	8.33 (0–25.3)	0.33 (0–17.3)	0.33 (0–14.7)	0.33 (0–16.7)	0.33 (0–16.7)	0.33 (0–16.0)	0.33 (0–17.3)
Slovakia	0.003	7.67 (0–24.0)	8.33 (0–25.3)	7.00 (0–24.0)	10.33 (0–26.0)	11.00 (0–28.0)	–	7.00 (0–24.0)	0.33 (0–21.3)	12.33 (0–28.7)	0.33 (0–20.0)	0.33 (0–15.3)	0.33 (0–18.7)	0.33 (0–21.3)	0.33 (0–20.0)
Bhutan	0.002	0.33 (0–17.3)	10.33 (0–26.0)	9.00 (0–25.3)	6.33 (0–24.7)	8.33 (0–25.3)	3.67 (0–22.0)	–	13.67 (0–29.3)	0.33 (0–14.0)	0.33 (0–16.0)	0.33 (0–16.0)	5.67 (0–23.3)	0.33 (0–20.0)	5.00 (0–23.3)
USA	0.000	0.33 (0–15.3)	7.67 (0–24.7)	9.00 (0–25.3)	7.00 (0–24.0)	15.67 (0–31.3)	0.33 (0–16.7)	11.67 (0–27.3)	–	2.33 (0–22.7)	0.33 (0–17.3)	0.33 (0–15.3)	4.33 (0–23.3)	0.33 (0–16.0)	0.33 (0–21.3)
Kenya	0.002	7.67 (0–24.7)	0.33 (0–17.3)	0.33 (0–20.7)	0.33 (0–20.7)	14.33 (0–31.3)	0.33 (0–16.7)	0.33 (0–15.3)	0.33 (0–16.7)	–	2.33 (0–19.3)	0.33 (0–17.3)	8.33 (0–25.3)	15.00 (0–30.7)	0.33 (0–14.7)
RSA	0.003	19.67 (2–36.0)	15 (0–30.7)	15.67 (0–31.3)	3.00 (0–20.7)	15.00 (0–31.3)	11.00 (0–27.3)	12.33 (0–28.7)	0.33 (0–14.0)	19.00 (0–36.0)	–	16.33 (0–32.7)	2.33 (0–22.0)	1.67 (0–22.0)	0.33 (0–28.7)
Chile	0.003	14.33 (0–30.0)	0.33 (0–17.3)	0.33 (0–17.3)	12.33 (0–28.0)	20.33 (2–37.3)	8.33 (0–24.0)	5.00 (0–23.3)	1.00 (0–22.0)	8.33 (0–25.3)	10.33 (0–26.0)	–	56.33 (39.3–72.7)	8.33 (0–25.3)	5.00 (0–23.3)
Ecuador	0.002	0.33 (0–16.7)	4.33 (0–22.7)	0.33 (0–21.3)	0.33 (0–16.0)	0.33 (0–22.7)	0.33 (0–17.3)	0.33 (0–21.3)	11.67 (0–28.0)	5.67 (0–23.3)	0.33 (0–14.7)	7.67 (0–24.0)	–	0.33 (0–20.00)	0.33 (0–20.7)
Australia	0.001	11.00 (0–26.7)	10.33 (0–26.0)	1.67 (0–22.0)	9.00 (0–25.3)	6.33 (0–26.0)	7.67 (0–24.7)	0.33 (0–18.7)	11.00 (0–27.3)	0.33 (0–19.3)	0.33 (0–20.0)	11.67 (0–27.3)	8.33 (0–25.3)	–	7.00 (0–23.3)
New Zealand	0.000	3.67 (0–22.7)	0.33 (0–19.3)	0.33 (0–16.0)	4.33 (0–23.3)	12.33 (0–28.7)	0.33 (0–14.7)	0.33 (0–21.3)	0.33 (0–20.7)	11.67 (0–27.3)	0.33 (0–15.3)	0.33 (0–22.0)	7.00 (0–24.7)	9.67 (0–25.3)	–

1 Donor or source populations are represented on the left while receiving or sink population are across.

2 *M* values are represented as the mode value. 95% Percentiles are indicated in parentheses.

### Mating type distribution

Both mating types were identified in all of the Northern Hemisphere populations. Isolates from Poland and the United States deviated from a 1:1 ratio of random mating with the *MAT1-2* idiomorph being significantly more common in Poland while the *MAT1-1* was more common in the United States (Table 3). In the Southern Hemisphere, all the isolates from Chile, Ecuador, Australia, and New Zealand were MAT 2. Although both mating types were found in Africa, only the population from Kenya showed evidence of a randomly mating population.

## Discussion

Clustering methods, based on multilocus microsatellite markers and applied to a broad collection of *D. septosporum* isolates, partitioned the isolates roughly corresponding to the geographical areas where they had been collected. These clusters also showed that the patterns of genetic diversity, population structure, and distribution of mating types in *D. septosporum* reflect anthropogenic activity relating to its distribution on non-native pines in the Southern Hemisphere. They also provide interesting insights into the establishment, reproduction and range expansion of *D. septosporum* on non-native and native trees in the Northern Hemisphere, particularly in Europe.

The European populations of *D. septosporum* were generally characterized by high levels of genetic diversity, high levels of migration between countries and the equal presence of both mating types. The collection of isolates from Austria, Hungary, Poland, the Czech Republic, Slovakia, and Romania constituted a single, large population, where most of the genetic variation (91%) is due to the large numbers of unique alleles present in the individual country collections. These populations, mainly on planted *P. nigra,* were genetically very similar, despite their occurrence in different countries. This can be explained by the fact that these countries border on each other and thus have no barriers to gene flow. Migration or range expansion of *D. septosporum* during the last 50 years in Europe*,* due to effective dispersal of asexual spores (conidia) and extensive movement of pine material (Tomšovský et al. 2012), has evidently resulted in the homogenization of allele frequencies in isolates from this region. This has apparently resulted in an even distribution of genetic structure of the pathogen in these countries. Such range expansion for an apparently native pathogen is not unusual and is well known for many other plant pathogens (Banke and McDonald 2005; Stukenbrock et al. 2006; Gladieux et al. 2008).

The fact that both mating types were found in more or less equal proportions in the populations of *D. septosporum* from Europe, and the high levels of genetic diversity observed, suggests that recombination between individuals is occurring. This interpretation is further supported by the fact that in most cases, the populations consisted of unique haplotypes and by the fact that the sexual state of the fungus has been recorded in several European countries (Kowalski and Jankowiak 1998; Bradshaw 2004) including those considered in this study. It is apparent that regular sexual recombination as well as population growth and expansion over time has gradually dissipated most of the evidence of introduced populations in Europe, which would be revealed from genetic bottlenecks and multilocus linkage disequilibrium (Dlugosch and Parker 2008).

The high genetic diversity of *D. septosporum* in Europe could be due to multiple introductions over time. However, based on the genetic evidence presented in this study, and others (Drenkhan et al. 2013; Kraj and Kowalski 2013), it is more likely that the pathogen is native to some areas of Europe although it has been, and still is, considered an alien invasive species and quarantine pest on this continent. The recent emergence of serious disease outbreaks and the observed range expansion of the pathogen might then be due to a combination of factors including climate change (Woods et al. 2005), increased availability of susceptible hosts arising from larger areas of planted forests and consequently, increased inoculum loads. A “spill-over” effect of the native pathogen onto newly available, susceptible hosts could then account for the high genetic diversity observed. A similar phenomenon has been observed in British Columbia (BC) where records of tree ring chronology suggest that *D. septosporum* has been present in this Canadian province since the 1800’s (Welsh et al. 2009). The recent severe epidemics in BC appear to be linked to an enormous increase of planting *P. contorta* var. *latifolia* in monocultures on sites supporting naturally mixed species as well as an increase in precipitation and temperature during the last decades, greatly favoring infections (Woods et al. 2005). The successful establishment and population build-up of *D. septosporum* in BC has evidently been facilitated by the high genetic diversity of the pathogen in native and non-native *P. contorta* var. *latifolia* stands (Dale et al. 2011) and the presence of both mating types (Groenewald et al. 2007).

Patterns of genetic diversity and the clustering of *D. septosporum* isolates from the Southern Hemisphere countries revealed the presence of three distinct evolutionary lineages that reflect the anthropogenic movement of pines into Africa, South America, and Australasia (Table S1). In the African cluster, the high migration rates from Europe, the high levels of genetic variability in the Kenyan and South African populations, and the presence of both mating types suggests that *D. septosporum* has most likely been introduced into these areas more than once. This is consistent with the long history of pine being moved into these countries (Poynton 1977; Richardson et al. 2007) that would have provided many pathways and opportunities for introduction of the pathogen (Lundquist 1987; Diekmann et al. 2002). A similar concordance between levels of genetic diversity and numerous putative introduction events has been found for the pine shoot and canker pathogen *Diplodia sapinea* on *P. radiata* in the Southern Hemisphere (Burgess et al. 2001), *Plasmopara viticola* populations in Europe (Gobbin et al. 2006) and the European race of *Gremmeniella albietina* var. *abietina* in North America (Hamelin et al. 1998).

Founder effects were evident in *D. septosporum* populations from Ecuador, Chile, New Zealand and Australia. The low levels of diversity and clonal structure that were observed in isolates from these countries suggest that these populations have lost alleles, which is consistent with their anthropogenic movement to new environments. This is common for introduced pathogens, for example, the pitch canker pathogen *Fusarium circinatum* (Wikler and Gordon 2000) and the chestnut blight fungus *C. parasitica* in south-eastern Europe (Milgroom et al. 2008). Clonality and shifts in the reproductive modes of populations are characteristic features for pathogens that have been moved from their areas of origin (Taylor et al. 1999). Both of these characteristics were apparent in the populations of *D. septosporum* from South America and Australasia in this study. In both regions, only the *MAT1-2* idiomorph was observed, which confirmed, using a larger sample size, the results of Groenewald et al. (2007).

Pathogen populations that have passed through bottlenecks resulting in lower levels of genetic diversity are thought to be at a disadvantage because of lost alleles that could potentially confer adaptive abilities to survive in their new environments (Sakai et al. 2001; Allendorf and Lundquist 2003). However, the low levels of genetic diversity and the presence of a small number of clonal lineages in the South American and Australasian clusters detected in this study showed that this has clearly not been a limiting factor for the successful establishment, spread and the occurrence of devastating *D. septosporum* epidemics in these Southern Hemisphere regions. In these cases, a small number of virulent genotypes have most likely thrived on the highly susceptible *P. radiata* under environmental conditions conducive for infection and spread exclusively via asexually produced spores. This would be similar to other introduced plant pathogens including, for example, *Phytophothora infestans* (Goodwin et al. 1994), *D. sapinea* (Burgess et al. 2001), and *Ceratocystis platani* (Engelbrecht et al. 2004).

Direct movement of *D. septosporum* between Chile and Ecuador was strongly supported by the genetic evidence in this study. Both countries shared common genotypes, all the alleles were identical and there was a lack of population structure (1% among-population variation) between these countries. DNB has been known in Chile since 1957 (Gibson 1972), but was reported from Ecuador only in 1983 (Evans and Oleas 1983). Given the genetic similarity of the *D. septosporum* populations in these countries and the strong evidence of directional migration observed using Migrate, it is likely that the pathogen was accidentally introduced from Chile into Ecuador. This is an excellent example of human-mediated movement because Chile is known to have shared *P. radiata* germplasm with Ecuador (F. Montenegro, pers. communication).

Results of this study clearly show that the *D. septosporum* population in New Zealand is clonal. This is consistent with previous investigations (Hirst et al. 1999; Groenewald et al. 2007) in which only one haplotype and a single mating type was found in samples covering a relatively large area. In the study by Hirst et al. (1999), collections from the 1960’s were identical to those made 30 years later. It is evident that the same clone continues to persist almost 50 years later. New Zealand is well known to have maintained very strict quarantine regulations for many years (http://www.maf.govt.nz/biosecurity-animal-welfare) and evidently, additional introductions of *D. septosporum* have not occurred.

It has been speculated that *D. septosporum* moved from New Zealand to Australia naturally across the Tasman Sea (Edwards and Walker 1978; Matheson 1985). Migrate analyses, however, suggest that the direction of migrants was most likely from Australia into New Zealand. The single haplotype present in New Zealand was also present in two locations sampled in Australia. However, the Australian samples included additional genotypes and unique alleles not found in New Zealand. This suggests that, despite rigorous quarantine regulations, there have been introductions from other sources into Australia, most likely from Europe and Africa. These would most likely have been human-mediated, due to the geographically isolated position of the continent, and they would most probably have occurred through the importation of pine germplasm.

The number of isolates from Kenya available for this study was limited. However, there were no shared genotypes between isolates from New Zealand and Kenya and only a small proportion (22%) of the alleles were common to the fungal populations from these areas. It was thus not possible to validate the hypothesis of Hirst et al. (1999) that *D. septosporum* in New Zealand or Australia originated in Kenya. Yet there is some evidence of low levels of migration from the African continent into Australasia.

The worldwide distribution of *D. septosporum* is an important example of human mediated, but unintentional movement of a plant pathogen. This movement has facilitated the establishment of *D. septosporum* in many areas of the world where pines are planted outside their natural ranges. Although available cultures from countries considered in this study varied in number, intriguing patterns of local range expansion and global spread emerged. There remains substantial opportunity to expand the knowledge gained in this study with larger numbers of isolates from areas that could not be sampled or where sampling was not optimal. In particular, it would be most interesting to consider the population genetics of isolates from North and Central America, which could provide intriguing insights into the possible area of origin of *D. septosporum*.

In the future, optimal climatic conditions, influenced dramatically by climate change, and the planting of susceptible trees in monocultures are likely to increase the potential for *D. septosporum* to infect new hosts, and to become established in new areas (Woods et al. 2005; Watt et al. 2009). This is apparently already occurring in areas such as British Columbia, Estonia, Finland, and Sweden (Woods et al. 2005; Brown and Webber 2008; Barnes et al. 2011; Drenkhan et al. 2013).

*Dothistroma septosporum* provides an outstanding model organism to study biological invasions because it has become established both in the Northern and Southern Hemisphere, where it can be viewed alternatively as a native pathogen or an alien invasive, influenced by planting practices, other human activities and climate change. Whereas populations of the pathogen in the Northern Hemisphere are genetically heterogenous and sexually recombining, those in the Southern Hemisphere, with the exception of Africa, have low levels of diversity and contain a single mating type gene. It will thus be important to monitor and prevent the further spread of different, possibly more virulent genotypes and opposite mating type genes, into these predominantly monoculture plantations of susceptible hosts in the Southern Hemisphere.

## Data accessibility

Microsatellite haplotype data can be found in DRYAD entry doi:10.5061/dryad.79364. Allelic frequencies of all the isolates are available in Table S2, supporting information.

## Supporting Information

Table S1 Dates for when pines (and more specifically, *P. radiata*) were introduced into some Southern Hemisphere countries. Dates are also recorded for when the susceptible species *P. radiata* was extensively grown in plantations and when the first reports and epidemics of Dothistroma needle blight (DNB) occurred.

Table S2 Allele frequencies of *Dothistroma septosporum* isolates from 14 different countries in six regions based on 12 microsatellite markers.
