# Supplementary material for: Population structure and diversity of an invasive pine needle pathogen reflects anthropogenic activity
Source: Ecol Evol. 2014 Sep 4;4(18):3642–61. doi: 10.1002/ece3.1200 (PMC4224538; doi:10.1002/ece3.1200)
Supplement: Table S1 — Dates for when pines (and more specifically, P. radiata) were introduced into some Southern Hemisphere countries. Dates are also recorded for when the susceptible species P. radiata was extensively grown in plantations and when the first reports and epidemics of Dothistroma needle blight (DNB) occurred. [file ece30004-3642-sd1.docx]

**Table S1** Dates for when pines in general and more specifically, *P. radiata*, were introduced into some Southern Hemisphere countries. Dates are also recorded for when the susceptible species *P. radiata* was extensively grown in plantations and when the first reports and epidemics of Dothistroma needle blight (DNB) occurred.

|  | **Pines introduced** | ***P. radiata* introduced** | **Extensive plantations of *P. radiata*** | **First reports of DNB** | **Epidemics of DNB** | **References** |
| --- | --- | --- | --- | --- | --- | --- |
|  |  |  |  |  |  |  |
| **Kenya** |  |  | 1945-1960 (1) | 1960 on *P. radiata* (from Tanzania) (2) | 1964 (3) | 1) Lavery & Mead 1998; 2) Gibson 1972; 3) Gibson et al 1964 |
| **Tanzania** |  |  |  | 1957 -young *P. radiata* Tanganyika (Tanzania) (1) | 1960 (1) | 1) Gibson 1972 |
| **Malawi** |  |  | 1928-1934 resumed again in 1955 (1) | 1940's on *P. radiata* (1) | 1962 (1) | 1) Gibson 1972; 2) Lavery & Mead 1998 |
| **Zimbabwe** |  | 1902 (3) | 1928-1934 (2) resumed again in 1955 (1) | Middle 1930's on *P. radiata* (4) | 1962 (1) | 1) Gibson 1972; 2) Lavery & Mead 1998; 3) Poynton 1977 4) Barnes 1970 |
| **South Africa** | late 17th century (1) | 1850 (2) | 1884 (2) | 1965 on *P. canariensis* (3) | 1984 - increased occurrence (4, 5) | 1) Poynton 1977; 2) Lavery & Mead 1998; 3) Gibson 1972; 4) Lundquist 1987; 5) Ivory 1994 |
| **Chile** |  | 1885 (1) | 1940's (1) | 1957 on *P. radiata* (2) | 1964 - 1965 (2) | 1) Toro & Gessel 1999; 2) Gibson 1972 |
| **New Zealand** | shortly before 1830 (1) | 1859 (2) | 1870's (3) | 1962 on *P. attenuata* x *P. radiata* but probably present since late 1950's (4) | 1966 (5) | 1) Richardson & Higgins 1998; 2) Hirst et al 1999; 3) Lavery & Mead 1998; 4) Gilmour 1967; 5) Gibson 1972 |
| **Australia** | 1770's with British colonization (1) | 1857 (1) | 1875 (2) | 1975 on *P. radiata* (3) | 1977 (3) | 1) Richardson & Higgins 1998; 2) Lavery & Mead 1998; 3) Edwards & Walker 1978 |

Barnes RD (1970) The prospects for re-establishing *Pinus radiata* as a commercially important species in Rhodesia. *South African Forestry Journal,* **72**, 17-19.

Edwards DW, Walker J (1978) *Dothistroma* needle blight in Australia. *Australian Forest Research,* **8**, 125-137.

Gibson IAS (1972) Dothistroma blight of *Pinus radiata. Annual review of Phytopathology,* **10**, 51-72.

Gibson IAS, Christiansen P, Munga F (1964) First observations in Kenya on a foliage disease of pines caused by *Dothistroma pini* Hulbary. *Commonwealth Forest Review,* **45**, 67-76.

Gilmour JW (1967) Distribution and significance of the needle blight of pines caused by *Dothistroma pini* in New Zealand. *Plant Disease Reporter,* **51**, 727-730.

Hirst P, Richardson TE, Carson SD, Bradshaw RE (1999) *Dothistroma pini* genetic diversity is low in New Zealand. *New Zealand Journal of Forest Science* **29**, 459-472.

Ivory MH (1994) Records of foliage pathogens of *Pinus* species in tropical countries. *Plant Pathology,* **43**, 511-518.

Lavery PB, Mead DJ (1998) *Pinus radiata*: a narrow endemic from North America takes on the world. In: Ecology and biogeography of *Pinus* (Richardson DM, ed) pp 432-449. Cambridge University Press, Cambridge, United Kingdom.

Lundquist JE (1987) Fungi associated with *Pinus* in South Africa. Part II. The Cape. *South African Forestry Journal,* **140**, 4-15.

Poynton RJ (1977) Tree planting in Southern Africa. Vol. 1 The Pines. *South African Forestry Research Institute*.

Richardson DM, Higgins SI (1998) Pines as invaders in the southern hemisphere. In: *Ecology and biogeography of Pinus* (Richardson DM, ed.) pp 450-473. Cambridge University Press, Cambridge, UK.

Toro J, Gessel SP (1999) Radiata pine plantations in Chile. *New Forests,* **18**, 33-44.
